# Adult patient communication experiences with nurses in cancer care settings: a qualitative study

**DOI:** 10.1186/s12912-022-00981-4

**Published:** 2022-07-26

**Authors:** Mukhlid Alshammari, Jed Duff, Michelle Guilhermino

**Affiliations:** 1 School of Nursing and Midwifery, University of Newcastle, Newcastle, Australia; 2 Nursing Department, College of Applied Medical Science, University of Hafr Al Batin, Hafr Al Batin, Saudi Arabia; 3 School of Nursing, Queensland University of Technology, Brisbane, Australia

**Keywords:** Pateint experience, Patient-centerd communication, Oncology, Cancer, Patient-nurse communication

## Abstract

**Background:**

The patient communication experience is an important outcome measure that guides quality improvements in healthcare settings specifically in cancer care. Therefore, this study aimed to explore the patient’s communication experiences with nurses in cancer care settings.

**Methods:**

Semi-structured face to face interviews were conducted with 21 participants who received cancer care at two Saudi Arabian tertiary healthcare facilities between Aug 2019 to Dec 2019. The study used a qualitative descriptive design. The interviews were audio-recorded and transcribed verbatim. Thematic analysis was used to analyse the data using six stages of Braun and Clarke.

**Results:**

Four major themes were identified and a total of eleven sub-themes. The major themes were; (1) The importance of patient-nurse relationships, (2) Providing appropriate information to patients, (3) Responding to patients emotional needs and (4) Verbal communication between nurses and cancer patients.

**Conclusions:**

Some participants felt that their communication with nurses was limited, but generally, most felt that communication was acceptable irrespective of barriers such as language, culture, religion, gender, workload and healthcare preferences. Participants drew a comparison between Saudi and non-Saudi nurses as well as between nurses and doctors communication skills. They felt that Saudi nurses had good communication skills, but non-Saudi nurses were more competent in some aspects such as kindness, politeness, respectful and non-verbal communication. They also felt that doctors were more accurate in their information than nurses.

## Background

A patient’s communication experience refers to the patient’s view of the communication and healthcare services they received from nurses and other healthcare providers during their hospitalisation [1]. The patient communication experience is an important outcome measure that guides quality improvements in healthcare settings. Improving patient communication is necessary to improve the quality and outcomes of healthcare as patients who report better communication experiences have been shown to have better health outcomes [2].

Patient-centred communication in cancer care model is focused on these patients’ communication experiences (R. M. Epstein et al., 2005) and consists of six factors that interact with one another to produce effective communication aimed at improving patients’ experiences and health outcomes. These factors are (1) exchanging information, (2) fostering healing relationships, (3) enabling patient self-management, (4) responding to patients’ emotions, (5) enabling patient decision-making and (6) managing uncertainty [3, 4].

In Saudi Arabia, a number of studies have been conducted to explore the experiences of nurses when communicating with patients who are undergoing cancer care [5, 6], while other studies have explored nurses’ perceptions and communication experiences in caring for patient populations other than those with cancer. These studies stated that expatriate nurses in Saudi cancer care settings face communication difficulties with patients as the majority are Saudi and speak the Arabic language. The nurses reported that these communication barriers adversely influenced patient care and their relationship with patients. However, no study has explored this topic from the point of view of these patients and their experiences communicating with nurses during cancer care in Saudi Arabia. This is an important gap in the literature given that expatriate nurses with different cultural backgrounds, languages and religions dominate Saudi Arabian healthcare [7, 8].

Evidence suggests that improved patient outcomes are more likely to be determined by patients’ experiences of communication rather than nurses’ communication experiences [9]. This underscores the need for an investigation of adult patients’ communication experiences in cancer care settings in Saudi Arabia using in-depth semi-structured face-to-face interviews.

## Methods

This study used a qualitative approach employing face to face semi-structured interviews with patients at cancer care settings.

## Study aim

This study aimed to explore adult patients’ communication experiences with nurses in cancer care settings.

## Study design

This study used a qualitative descriptive design to explore the patient–nurse experiences of 21 patients with cancer.

## Settings

This qualitative study was conducted at two referral healthcare facilities, King Faisal Specialist Hospital (KFSH) and King Fahad Medical City (KFMC), in Riyadh, Saudi Arabia. The facilities provide specialised services for various conditions, including cancer, and are the major cancer referral hospitals in Saudi Arabia. Together, they provide cancer care for over 55,000 patients annually as either inpatients or outpatients [10, 11].

## Participants

Convenient sampling was used to recruite patients aged between 18 and 90 years who were receiving cancer care at either the KFMC or KFSH. Patients receiving pallaitve care were excluded from the study. An advertising flyer providing information to potential participants was placed in the nursing stations and clinics at each facility. The researcher obtained written informed consent from the participants prior to the interviews. The participants were allowed to have family member (s) present in their interviews if preferred.

## Data collection

This study used semi-structured face-to-face interviews, which allowed for unplanned additional follow-up questions and a deeper understanding of patients’ communication experiences with the nurses at KFSH and KFMC [12, 13]. The research guide was developed based on previous literature, theories and conceptual frameworks [4, 13, 14]. The interview guide focused on six core sections, namely, (1) the patient–provider relationship, (2) exchanging information, (2) patients’ emotional needs, (4) help managing uncertainty, (5) involving patients in decision-making and (6) enabling patient self-management (Table 1). The in-depth interviews were conducted in a private meeting room at each hospital. All the interviews were scheduled to last approximately 30 min to one hour [13]; however, the interviews continued until the research team felt they had obtained a deep understanding of the issues [15]. The sample size was considered sufficient when data saturation was met and the researcher was not collecting any new information from the interviews [16].

**Table 1:** Semi-structured interview guide

1. Please, tell me about your experience in terms of exchanging information with nurses?
2. Tell me about your relationship with nurses?
3. What is your experience in making decisions concerning your condition?
4. How well did the nurses express interest in your emotional wellbeing?
5. What support did nurses provide to you in managing self-care?
6. What was your experience regarding how nurses helped you in dealing with the uncertainty of your illness?
7. Tell me about your overall experience with the healthcare you received from nurses?

## Data analysis

**Fig. 1:**
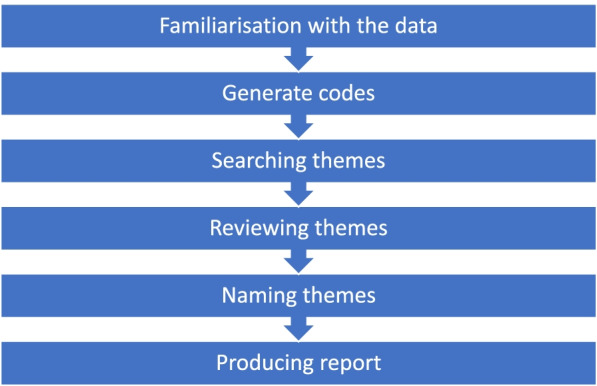
Six stages of thematic analysis by Braun and Clark

The recorded data were transcribed verbatim. Figure 1 illustrtared a thematic analysis that was applied based on the six stages outlined by V Braun and V Clarke [17]. First, the transcribed data were read and reread so that the researcher could become familiar with it. After that, the data were labelled and orgiansed into meaningful groups (codes) with full attention given to all data items. For instance, the initial codes were formed by coding features of the data in a systematic way that are relevant to the research aim and collated. Then, these codes were grouped into preliminary subthemes and themes. The themes were reviewed by rereading the data several times and checking if the theme fit the coded. A thematic map was developed where the themes were defined and named.

This approach was selected because it is flexible and enables themes to be identified from large bodies of data [18].

## Rigour

To maintain rigour, the stduy followed Guba and Lincoln’s trustworthiness criteria [19]. The trustworthiness of the study was ensured by the member checking with the participants (participants were offered the opportunity to review the thematic map with quotes) and conducting debriefing sessions with co-authors to obtain reflective feedback. An audit trail in the form of a researcher diary was maintained to document the interpretations and conclusions by the research team. The research team confirmed the accuracy of the primary data. In addition, the study was reported with sufficient depth of description to ensure appropriate transferability to another context [19].

## Results

In this study, 21 participants from KFSH and KFMC were interviewed individually. Seven of these participants requested that a member of their family accompany them during the interview, and seven family members participated in the interviews with the participants (Table 2). Of the 21 participants who completed the interviews, 16 were men, and five were women. The interviews were conducted over four months by the first author (August–December 2019).

**Table 2:** Particpants demographical information

	**Gender**	**Age**	**Geographical Location**	**Nationality**	**Accombined by family member**
Participant 1	Male	25	City	Saudi	No
Participant 2	Male	28	Rural	Saudi	No
Participant 3	Female	65	City	Saudi	Yes (Her daughter)
Participant 4	Male	26	City	Non-Saudi	Yes (His father)
Participant 5	Male	52	City	Saudi	No
Participant 6	Male	37	City	Saudi	No
Participant 7	Male	68	Rural	Saudi	No
Participant 8	Male	55	City	Saudi	No
Participant 9	Male	42	City	Saudi	No
Participant 10	Male	66	City	Non-Saudi	No
Participant 11	Male	54	City	Saudi	No
Participant 12	Female	74	City	Saudi	Yes (Her daughter)
Participant 13	Male	27	City	Saudi	No
Participant 14	Female	70	City	Saudi	Yes (Her daughter)
Participant 15	Female	35	Rural	Saudi	Yes (Her sister)
Participant 16	Male	39	Rual	Saudi	Yes (His brother)
Participant 17	Male	60	City	Saudi	No
Participant 18	Male	22	City	Saudi	No
Participant 19	Female	75	Rural	Saudi	Yes (Her son)
Participant 20	Male	42	City	Saudi	No
Participant 21	Male	30	City	Saudi	No

An analysis of the transcribed interviews resulted in the identification of four major themes: nurse–patient relationships, providing information, responding to emotions and verbal communication (Table 3).

**Table 3:** Underpinning themes and sub-themes

Underpinning themes	Sub-themes
Patient-nurse relationships	- Responsivnes
	- Kindness, respectful and politeness
	- Trust
Providing information	- Exchanging information
	- Decision making
Responding to emotions	- Doubts, fears and uncertainties
	- Non-verbal support
	- Psychological support
Verbal communication	- Language barriers
	- Communication strategies

### Theme 1: Patient–nurse relationships

The participants reported that nurses responsivness, kindness or politeness and trustworthyness played an important role in forming strong patient–nurse relationships.

#### Responsivness

Patient response was an important factor in establishing patient–nurse relationships. Several participants described their response and interaction with the nurses as good because they felt they could communicate with the nurses freely. The participants also described the importance of other responses such as smile, saying that a nurse’s smile was enough to provide them with adequate support and assurance. However, a number of the participants had different opinions and reported little different responses with the nurses. For example, some participants commented that there was little response and interaction as the nurses ‘merely give the medication and leave’.

‘Yes, the nurses interacted with me and reassured me... They are excellent... I can communicate with them freely... They always smile at me.’ (Participant 3).

‘There is little response. They give me the medicine and go, then I only see them on the second day of the second dose.’ (Participant 6).

The participants generally reported more positive responsiveness with the Saudi nurses. In particular, some of the participants explained that the Saudi nurses introduced themselves to patients while the non-Saudi nurses did not. The participants noted that they liked the Saudi nurses’ approach to response because they were shared the same culture and spoke the same language and could therefore understand each other.

Gender was also found to impede response of the nurses as the female participants found interaction with the male nurses to be awkward. Specifically, some of the female participants indicated shyness towards the Saudi male nurses, which limited their ability to interact effectively with the nursing staff*.* This awkwardness was perceived as reciprocal by some of the participants*.*

‘Saudi nurses generally introduce themselves by their names... They understand me, and I understand them... Some of the foreigners don’t introduce themselves... I think introducing themselves would be very good [as it would] reassure patients.’ (Participant 8).

‘I do not ask the nurses much, first because I feel that [the nurse] is embarrassed with me as a Saudi and, second, because I am embarrassed with Saudi men... Yes, even he feels embarrassed, not only us. In terms of response, they does not response or interact much as they comes and supervises the device and leaves.’ (Participant 15).

#### Kindness, respectfulness and politeness

The participants drew comparisons between the foreign and Saudi nurses. Some of the participants felt that the foreign nurses should be commended for their kindness, respectfulness and politeness, while the Saudi nurses were perceived to lack these qualities. A few of the participants suggested that the Saudi nurses should learn these behaviours from the expatriate nurses.

‘I suggest that the Saudi’s learn kindness, respect and politeness better... The nurses here who are European and non-Saudi are professional staff whereas we Saudis are so far different in this matter... I wish that the Saudis could have the same attention as the foreigners.’ (Participant 18).

The participants indicated that the respect they had for the nurses was reciprocal. One participant further described the patient–nurse relationship as a relationship of respect, while another explained their respect for the nurses in terms of the nurses’ kindness and politeness.

‘If you respect [the nurses], they respect you... My relationship with the nurses is a relationship of respect and appreciation.’ (Participant 7).

‘Some non-Saudi nurses here respond in a beautiful way. Even when they give me an injection, they say sorry, and they are not supposed to be sorry. This is the height of kindness... The nurses help me all the time and always check on me, which is why I respect them, and I feel that they are more professional in terms of kindness, politeness, helping me and checking on me all the time.’ (Participant 21).

#### Trust

Some of the participants described their relationships with the nurses as trustworthy because they could depend on the nurses when receiving care. They described their dependence and reliance on the nurses, which they indicated was instrumental in developing trust. In addition, the participants noted that this trust was based on the fact that the nurses knew what they were doing, and this was their speciality. In contrast, other participants commented that they preferred to ask the doctors about their health rather than the nurses because they believed the doctors provided more accurate information.

‘I depend on the nurses for many things... This is their specialty, and I take the nurses’ words very seriously.’ (Participant 13).

‘I give the nurses a piece of my trust, not my full trust... I do not ask the nurses about these things. I try to ask the doctor because I do not want to hear an answer that they are not sure of. It causes me tension, so I want to hear a definite answer.’ (Participant 16).

### Theme 2: Providing information

Providing information to patients is important as patients should know every detail of their health status. This can be achieved through an exchange of information between healthcare providers and patients about the patients’ health to facilitate their participation in their health.

#### Exchanging information

The ability of the nurses to exchange information with the participants was heavily influenced by the nurses’ workloads. Many participants reported that the nurses were too busy to answer their questions, while others commented that the information and quality of the responses they received from the nurses appeared to be dependent on the nurses’ attitudes as they answered some questions and not others. These participants were told to wait for the doctors to obtain information. These participants could not obtain all the necessary information from the nurses, so they sought it from the doctors instead.

‘There is communication depending on the number of patients and nurses. As you know, the hospital is busy – and the nurses as well... The nurses refuse to answer some candid questions.’ (Participant 9).

‘If I ask the nurses some questions, they respond that when the doctor comes, [I should] talk to him... I can’t get the necessary information from the nurses, but I can get it from the doctors.’ (Participant 1).

Some of the participants who exchanged information with the nurses reported that they received a sufficient amount of information from the nurses. Most of these participants were comfortable asking the nurses for information. They stated that the nurses were able to explain what they wanted to know.

‘The amount of information [received from the nurses] is enough for me.’ (Participant 4).

‘The nurses explain the things that I want to know to me, for example, what this treatment does... The nurses give me information about this disease and how to deal with it.’ (Participant 15).

#### Decision-making

The participants rarely discussed their medical conditions or treatment with the nurses. They viewed the nurses as treatment providers who carried out the instructions of the doctors. In addition, the nurses were seen to provide care and treatment but did not share or ask for the patients’ opinions on that care.

‘Sometimes, if I ask the nurses, they do not give me an answer; the nurses mostly provide treatment. Therefore, I do not usually ask the nurses about my condition or care choices; [instead], I try to ask the doctor. Also, I do not want to hear different answers; I want to hear an answer that can help me make decisions.’ (Participant 16).

‘The nurses give me medicine immediately. They do not discuss the treatment with me, and they do not ask me [my opinion]. But sometimes they tell me what the benefit of this treatment is and what it will do.’ (Participant 1).

A few participants did, however, discuss their treatment options, care and decision-making with the nurses. These participants noted that the nurses were well educated and competent and provided patients with the necessary brochures and resources to enable them to participate in the decision-making regarding their health.

‘The nurses are very well educated, and they help me and give me resources to help me make decisions... I usually ask the nurses, are there people who have recovered? After how many sessions?... What can this medication do, and what do you think is best for my health?... What should I avoid?’ (Participant 4).

### Theme 3: Responding to emotions

According to the participants, some of the nurses responded to their doubts, fears and uncertainties by providing psychological and non-verbal support, while other nurses did not respond to patients’ emotions due to barriers such as cultural and religious differences.

#### Doubts, fears and uncertainties

Very few of the study participants discussed their doubts, fears and uncertainties with the nurses. Overall, there was a tendency for the participants to discuss their fears with their doctors and families rather than the nurses. The participants suggested that the nurses did not provide them with an opportunity to share their fears, and they mostly preferred to share their feelings with their families instead. However, one participant reported that they shared their doubts and fears with the nurses, and they received reassurance from them.

‘No, the nurses do not talk about [my] fears and uncertainties; they just talk about the procedures they are doing, such as vital signs, but the doctors do that... The nurses do not give me a chance to do that... I like to discuss it with my family.’ (Participant 1).

‘The nurses are positive... They told me not to be afraid, everything will be fine. They also told me about the side effects of the drugs and taught me how to deal with them, which gave me a kind of relief.’ (Participant 4).

#### Non-verbal support

The participants described the importance of non-verbal support in boosting their morale. They perceived the non-Saudi nurses as more competent in terms of non-verbal communication such as smiling. They saw this form of communication as relieving the stress of their disease. The participants also commented that the Saudi nurses needed to attend courses on how to smile at patients at the right times.

‘There is no comparison when someone comes to me [and they are] smiling... That is enough to make me [feel] comfortable... Their non-verbal communication is excellent... That is what the patient wants, a smile on their face or a gentle response... I think the Saudi nurses need to attend courses on how to smile and do other positive non-verbal communication.’ (Participant 19).

#### Psychological support

Interestingly, the participants were relatively divided regarding the nurses’ ability to respond to patients’ emotions. Some of the participants noted that the nurses were excellent and provided psychological support to overcome their tension regarding their disease and associated fears and emotions. Other participants stated that the nurses were sensitive towards their feelings and expressed care, interest and concern regarding their overall well-being because psychological support was not perceived as the role of nurses, which made the patients hesitant to talk about their feelings.

‘Overall, the nurses are all excellent; sometimes there is a delay in some services, but in terms of psychological support, they are good... Most of the time, the nurses are positive. They support me psychologically to overcome this disease.’ (Participant 4).

‘No one cares. The nurses come and do their job and leave... There is no care about other people’s feelings... The disease is different because my problems have nothing to do with their speciality and have nothing to do with nursing. This is my problem, so it is not possible to talk to the nurses about it.’ (Participant 13).

The participants noticed a difference between the nurses’ ability to respond to emotions and their nationality. It was reported that not all the nurses had a sufficient understanding of the patients’ feelings to support them psychologically due to a lack of understanding of the unique Saudi culture. Religion also played an important role in the psychological support and care of patients. The participants perceived that the Saudi nurses were more understanding and aware of their religion. The Saudi nurses were also acknowledged for encouraging the patients to pray and easing the patients’ fears and distress.

‘Sometimes the Saudi nurses provide me with psychological support. If I complain, they understand what I want and what I don’t want, but some of the foreigners, no matter how I talk to them, do not respond like the Saudis.’ (Participant 8).

‘When I want to pray in the room, the Saudi nurses close the curtain and say to me that you can pray. They care about that... They encourage me to pray and [say] that everything will be fine, and they encourage me to trust the treatment and [say that] I will be fine.’ (Participant 5).

### Theme 4: Verbal communication

A number of the participants perceived verbal communication to be very important. Arabic was the preferred language of some of the participants, but others indicated that they could speak Arabic and English and had no problem speaking either language. Despite this, the participants who only spoke Arabic reported using several methods to overcome the communication barriers.

#### Language barriers

Language barriers hindered the patients’ communication with the nursing staff. Many of the participants stated that they were unable to understand the nurses who did not speak Arabic as the participants did not speak English. The few participants who could speak English did not report any problems in terms of communication.

‘Yes, it is a problem. I do not understand their conversation sometimes and what they want from me, and they do not understand what I am saying... I do not have a language to communicate with them because most of them are from abroad.’ (Participant 15).

‘I can communicate with the nurses effectively because I can speak English... The nurses sometimes explain the many things that I need to know to me.’ (Participant 11).

#### Communication strategies

Some of the participants reported overcoming the language barriers by using some communication strategies such as sign language and other non-verbal cues to communicate with the foreign nurses, while others indicated that they used an interpreter to overcome the language barriers.

‘When expatriate nurses do not understand my words, I ask Arabic healthcare providers or the formal hospital translator to talk to the expatriate nurses, and they translate for me... Sometimes I use sign language to explain something or deliver my idea. Most of the time they understand my sign language [laughs]... Currently the only obstacle is language. I communicate with them normally, and I mean I can communicate with them, but sometimes they do not understand me.’ (Participant 2).

Some of the participants reported that employing more Arabian nurses in the hospital would be beneficial to them. Alternatively, it was recommended that foreign nurses learn Arabic. Training in patient communication and non-verbal communication was also advised. One participant commented on the need for foreign nurses to be aware of Saudi Arabian religiosity.

‘The nurses must learn Arabic because not all people know English or have facilities to understand the language... Employing more Arabic nurses is the way to solve this problem... Non-Saudi nurses who do not speak Arabic should learn the Arabic language to communicate with us effectively.’ (Participant 12).

## Discussion

This study’s findings provide insights into the experiences of patients and their families in terms of communication with nurses in cancer care settings. It provides rich information regarding cancer patients’ communication experiences with nurses based on patient-centred communication theory.

### Nurse–patient relationships

The participants had varying experiences of nurse–patient relationship. For instance, some shared positive accounts of their communication with the nurses caring for them, others reported little communication with the nurses. The nurses who spoke the same language as the patients communicated effectively with them. Notwithstanding, some of the participants reported that effective communication with the nurses was restricted by gender differences despite the use of the same language. This finding is similar to that of an international study conducted in Jordanian cancer settings [20]. The study revealed communication restrictions due to gender differences as the female patients were more likely to prefer female healthcare providers and communicated with them to a greater extent.

The participants in this study indicated contrasts between the Saudi and non-Saudi nurses with regard to their levels of kindness, respect and politeness. In particular, although the Saudi nurses spoke the same language, some of the participants thought that the non-Saudi nurses were more professional in terms of respect, kindness, pleasantness, friendliness and helpfulness and indicated that the non-Saudi nurses checked on patients frequently. This could influence the nurse–patient relationship and increase patient satisfaction despite some communication barriers. A study by Atallah, Hamdan‐Mansour, Al‐Sayed and Aboshaiqah (2013) explored patient satisfaction with nurses’ quality of care in general patient populations and found that the non-Saudi or expatriate nurses were polite and kind. However, the study did not compare Saudi and non-Saudi nurses with regard to kindness, respect and politeness. The participants in this study felt that more education and training on cancer care was needed to develop the Saudi nurses’ competence in terms of kindness, respect and politeness and the need to always check on patients.

### Providing information

Nurses play an important role in providing information to patients. This information is used to assist patient decision-making and helps provide patients with an understanding of their disease, treatment plan and the potential side effects of their treatment and medication. Our findings suggest that despite good nurse–patient communication in general, the exchange of information and patient decision-making were influenced by the workload, gender and nationality of the nurses and the healthcare recipients’ preferences. Previous research exploring the perceptions of nurses in different Arab countries obtained similar results regarding the influence of workload [21], gender differences [20] and the different nationalities, languages and cultures of nurses [22] on communication. However, the preferences of healthcare recipients were a new finding in the Saudi Arabian context.

Most of the participants in the current study drew a comparison between the doctors’ communication and that of the nurses and described the physician’s communication as better than that of the nurses because the information provided by the physicians was perceived as more accurate. In addition, the nurses limited their opportunities to share information with the patients by telling them to wait for the physicians when the participants sought information about their health. This created the perception that the nurses were less important than the doctors, and the doctors were responsible for sharing important health information. This in turn led the participants to believe that they could only discuss their medical issues and treatment with their physicians. A possible explanation for this situation could be nurses’ fear of responsibility from hospital administration as they deliver sensitive information about cancer disease because they consider physician to be the responsible person to deliver these information to cancer patients. A study in Iran found that the person who is primarily responsible for delivering sensitive information to patients in cancer care settings is the physician [23].

### Responding to emotions

Nurses play an important role in boosting patient morale and responding to emotions. Some of the participants in this study indicated that they rarely discussed their emotions with the nurses and preferred to discuss their emotional issues with their families. The strong relationships between patients and their families in Saudi Arabia could explain this. A systematic review that included five studies from various countries found that the cancer patients generally did not like to share their emotions with nurses but preferred to share them with their families [24].

A few of the participants in this study reported receiving adequate psychological support from the nurses. Notably, most of the participants felt that the expatriate nurses did not understand their experiences because of cultural and language barriers [22], while the Saudi nurses helped the patients practise their religion and pray during hospitalisation because they shared the same religion. This spiritual support was seen by the participants as a form of psychological support. A similar study exploring Saudi nurses’ perceptions of Saudi patients found that prayer time is important to most patients, and the nurses therefore avoided disturbing the patients during these times [6].

### Verbal and non-verbal communication

Language barriers during cancer care in Saudi Arabia are the most common patient–nurse problem, with most patients being responsible for causing other problems. A study explored the barriers to nurses’ perceptions of end-of-life care and found that the language barrier was the main problem facing nurses working daily in cancer care settings in Saudi Arabia [25]. Similarly, many of the participants in this study reported they could not understand everything the expatriate nurses said, but they could understand the issues if they were conveyed by a hospital interpreter, Arabic healthcare providers or family members.

Some of the participants reported better nonverbal communication, such as smiles, from the non-Saudi nurses compared to the Saudi nurses. They felt that more education and training was needed to improve Saudi nurses’ competence in non-verbal communication with cancer patients. This is understandable from a patient perspective as non-verbal communication can be seen as a form of assurance for many patients [26].

### Implications for future practice and research

Governments and healthcare facilities should be encouraged to employ more nurses or to manage the patient distribution to nurses (patient ratio) to reduce nurses’ workloads. It would be beneficial for the Saudi health service to employ more Arabian nurses in cancer care settings as cancer patients need comprehensive communication to express their feelings and make suitable decisions regarding their health [27]. If this were not possible, the health service should develop a specific program to help foreign nurses learn the Arabic language, religion and culture before their arrival in Saudi Arabia. This program should be reviewed by the health ministry and include a first-year competency evaluation for new employees.

Many of the participants in this study reported good-to-excellent communication with the Saudi nurses because they shared the same language, culture and religion; however, many recommended that educational programs or information-sharing sessions be implemented for Saudi nurses working in cancer care settings to inform them of possible challenges in the care of patients with cancer and to develop their competences in terms of politeness, kindness, respect, the importance of checking on patients regularly and non-verbal communication such as smiling. The study further recommend that education stressing the importance of open and honest communication with patients and their families be provided for all nurses working in cancer care in Saudi Arabia. This will help build cancer patients’ trust in nurses, encourage nurses to become more emotionally involved with patients and motivate patients to participate in their health.

## Conclusion

This study explored patients communication experiences with nurses in cancer care. To our knowledge, this is the first study that explored patients communication experiences from patients perspectives in Saudi cancer care settings. Some participants felt that their communication with nurses was limited, but generally, most felt that communication was acceptable irrespective of barriers such as language, culture, religion, gender, workload and healthcare preferences. Participants reported their different experiences communicating with Saudi and non-Saudi nurses. They felt that Saudi nurses had good communication skills, but non-Saudi nurses were more competent in some aspects such as kindness, politeness, respectful and non-verbal communication. They also felt that doctors were more accurate in their information than nurses.

## Strengths and limitations

A strength of this study was the semi-structured interviews as these allowed the participants to reflect on important issues and to share their previous and current nurse–patient communication experiences. Nevertheless, all the participants were receiving cancer care, so the findings of this study may not reflect the experiences of all patients in Saudi Arabia.

## Data Availability

The datasets generated during and/or analyzed during the current study are not publicly available due confidentiality of participants but are available from the corresponding author on reasonable request.

## Abbreviations

KFSH: King Faisal Specialist Hospital
KFMC: King Fahad Medical City
